# Calming the Nerves via the Immune Instructive Physiochemical Properties of Self‐Assembling Peptide Hydrogels

**DOI:** 10.1002/advs.202303707

**Published:** 2023-11-29

**Authors:** Negar Mahmoudi, Elmira Mohamed, Shiva Soltani Dehnavi, Lilith M. Caballero Aguilar, Alan R. Harvey, Clare L. Parish, Richard J. Williams, David R. Nisbet

**Affiliations:** ^1^ Laboratory of Advanced Biomaterials the John Curtin School of Medical Research Australian National University Canberra ACT 2601 Australia; ^2^ ANU College of Engineering & Computer Science Australian National University Canberra ACT 2601 Australia; ^3^ The Graeme Clark Institute The University of Melbourne Melbourne VIC 3010 Australia; ^4^ Department of Biomedical Engineering Faculty of Engineering and Information Technology The University of Melbourne Melbourne VIC 3010 Australia; ^5^ School of Human Sciences The University of Western Australia and Perron Institute for Neurological and Translational Science Perth WA 6009 Australia; ^6^ The Florey Institute of Neuroscience and Mental Health The University of Melbourne Parkville Melbourne VIC 3010 Australia; ^7^ IMPACT School of Medicine Deakin University Geelong VIC 3217 Australia; ^8^ Melbourne Medical School Faculty of Medicine Dentistry and Health Science The University of Melbourne Melbourne VIC 3010 Australia

**Keywords:** bioactive scaffolds, foreign body reaction, neural regeneration, self‐assembling peptide‐based hydrogels, tissue engineering

## Abstract

Current therapies for the devastating damage caused by traumatic brain injuries (TBI) are limited. This is in part due to poor drug efficacy to modulate neuroinflammation, angiogenesis and/or promoting neuroprotection and is the combined result of challenges in getting drugs across the blood brain barrier, in a targeted approach. The negative impact of the injured extracellular matrix (ECM) has been identified as a factor in restricting post‐injury plasticity of residual neurons and is shown to reduce the functional integration of grafted cells. Therefore, new strategies are needed to manipulate the extracellular environment at the subacute phase to enhance brain regeneration. In this review, potential strategies are to be discussed for the treatment of TBI by using self‐assembling peptide (SAP) hydrogels, fabricated via the rational design of supramolecular peptide scaffolds, as an artificial ECM which under the appropriate conditions yields a supramolecular hydrogel. Sequence selection of the peptides allows the tuning of these hydrogels' physical and biochemical properties such as charge, hydrophobicity, cell adhesiveness, stiffness, factor presentation, degradation profile and responsiveness to (external) stimuli. This review aims to facilitate the development of more intelligent biomaterials in the future to satisfy the parameters, requirements, and opportunities for the effective treatment of TBI.

## Graphical abstract

Modulating the physicochemical properties of self‐assembled peptide (SAP) hydrogels such as charge, hydrophobicity/hydrophilicity, cell adhesiveness, stiffness, therapeutic/growth factor presentation, and responsiveness to (external) stimuli can reduce neuroinflammatory responses and enhance neuroprotection in the treatment of traumatic brain injury.

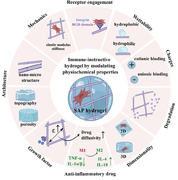

## 1 Introduction

Due to a lack of effective treatments, traumatic brain injury (TBI) is one of the leading causes of long‐term disability and death worldwide, resulting in an enormous socio‐economic burden to patients, patients’ families and broader society.^[^ ^1^, ^2^ ^]^ TBI has a complex prognosis, as it includes any physical event resulting in insult – typically a primary (direct) mechanical injury and secondary (indirect) insult to the surrounding parenchyma.^[^ ^3^ ^]^ The neurological outcome depends on the extent of TBI at the time of the injury which can be associated with the nature and mechanism of the injury, e.g., a high‐speed collision might result in more severe injury compared to a minor fall.^[^ ^4^, ^5^ ^]^ The population can also be variably affected, noting severe injuries are more frequent among males and people over 60 years old.^[^ ^6^ ^]^ The severity of TBI in patients are most commonly rated using the clinical Glasgow Coma Scale (GCS) which divides the injuries into mild (GCS 14 to 15), moderate (GCS 9 to 13), and severe TBI (GCS 3 to 8) and spans a spectrum of symptoms as mild as brief confusion through to death.^[^ ^7^, ^8^ ^]^ Complex secondary brain injury is difficult to treat long term, as it includes disturbance of ion homeostasis, inflammation, demyelination, cell death, excitatory toxicity, and oxidative stress.^[^ ^9^ ^]^ The role of the inflammatory response post‐TBI is complex because it can promote both beneficial clearance of debris and help cordon off an area of injury, but it can also be detrimental leading to neuronal death and chronic neurodegeneration. Yet, despite the numerous efforts focused on treatment strategies for neural loss, no current medical intervention can significantly and consistently improve the spontaneous restoration of neurological functions. This is due in part to the structural complexity of the tissue.^[^ ^10^, ^11^ ^]^ The formation of a cyst cavity post‐TBI is common. This failure of regenerating functional tissue in and around the injury is partly attributed to the unfavourable environment and the absence of, or damage to, the surrounding extracellular matrix (ECM). The ECM performs essential functions in the brain and further provides a 3D space for cells to orientate themselves within, allowing for complex signalling network to control their function once established.^[^ ^12^ ^]^ Treatments often fail due to an inability to replace this ECM structure, coupled with a lack of a therapeutic delivery system that can deliver sustained release of drug/therapeutic molecules at effective/therapeutic concentrations, in the relevant location, and for the appropriate length of time.^[^ ^13^ ^]^

In part, the regenerative capacity of tissues is determined by the plasticity and robustness of the ECM, and disruption thereof can cause significant adverse effects.^[^ ^14^ ^]^ Rapid changes to the ECM occurring during injury, or more gradual degradation due to neurodegenerative disease, means the natural ECM cannot effectively support cells within the affected volume of tissue. This lack of support may hinder the infiltration and organisation of regenerative cellular elements in the local region.

**Figure 1:**
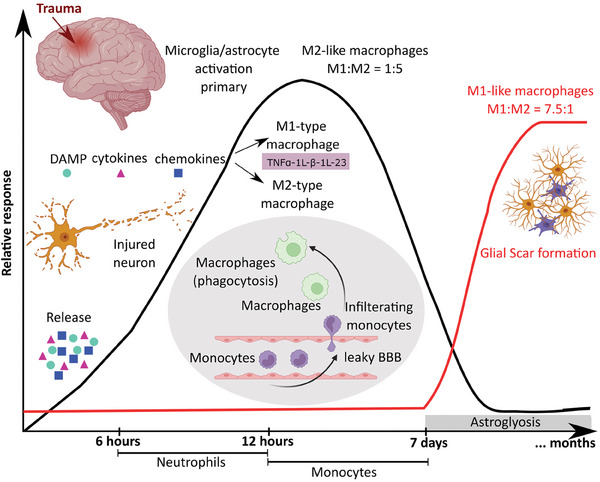
Schematic illustration showing the temporal cellular infiltration response after TBI. 1) The release of damage‐associated molecular patterns. 2) Microglia and astrocytes initiating a cascade of immune events. 3) Neutrophils infiltrating the lesion sites within 24 h post injury and contributing to inflammation. 4) Release of matrix metalloproteinase (MMPs) and generation of cytotoxic amounts of reactive nitrogen species and reactive oxygen species (ROS). 5) Activation and polarization of microglia. 6) Shifting the ratio of M1:M2 microglia from 1:5 (day 1) to 7.5:1 (day 7) following injury.^[^ ^31^, ^32^ ^]^ Created with BioRender.com.

Substantial breakthroughs have been made in understanding central nervous system (CNS) pathology in response to injury, which are important to consider when designing effective biomaterial interventions.^[^ ^15^ ^]^ Following a primary injury, secondary damage is provoked through a complex and ongoing cascade of cellular and molecular events, including further axonal degeneration, demyelination, neuroinflammation, oxidative stress/free radical formation, impaired metabolism, mitochondrial and synaptic damage, cerebral edema, and disruption of the blood–brain barrier (BBB).^[^ ^16^, ^17^ ^]^ In response to injury, dynamic multicellular interactions (**Figure** **1**) effectively isolate adjacent viable neural tissue and allow recruited inflammatory cells to resolve potentially harmful elements.^[^ ^18^, ^19^, ^20^ ^]^ Following TBI, microglia and astrocytes initiate a series of immune events governed by the release of damage‐associated molecular patterns (DAMPs).^[^ ^21^, ^22^ ^]^ Neutrophils are the first leukocytes that infiltrate lesion sites, typically within 24 h post injury. Neutrophils are believed to contribute to inflammation by further destabilizing the BBB with the release of matrix metalloproteinase (MMPs) and generating cytotoxic amounts of reactive oxygen species (ROS), reactive nitrogen species and enzymes such as inducible nitric oxide synthase (iNOS). The inflammatory cytokines produced by leukocytes, astrocytes, microglia, and neurons induce local and systemic immune responses.^[^ ^23^ ^]^ This causes further neuronal cell death and attracts more immune cells into the lesion further exacerbating the inflammatory response. Post‐injury changes in the characteristics of the extracellular space are also critical.^[^ ^24^ ^]^

Activation and polarization of microglia, the resident CNS macrophage, is integral to the response of brain injury^[^ ^25^ ^]^ Macrophages/ and microglia exist as pro‐inflammatory (M1) and anti‐inflammatory/pro‐healing (M2) phenotypes.^[^ ^26^, ^27^, ^28^ ^]^ The latest research indicates that the majority of the recruited microglia/ and macrophages at the site of injury have mixed M1‐ and M2‐ like activation profiles^[^ ^29^ ^]^ Within one week of injury, there is a shift from M2‐like microglia toward M1‐like microglia^[^ ^30^ ^]^ As a result, the ratio of M1:M2 microglia on day 1 shifts from 1:5 to 7.5:1 on day 7 following injury^[^ ^31^ ^]^ Several factors induce M1‐/M2‐like polarization, such as injury severity, location within grey or white matter, sex, or aging. For efficient tissue repair post‐TBI, both M1‐like and M2‐like functional responses are required. Despite the essential role microglia plays in the recovery of the nervous system from injury, they can also contribute to sustained inflammation that can limit recovery and drive chronic disease processes. Migration of activated microglia and astrocytes to the injury site is a hallmark of neuroinflammation resulting in the release of pro‐inflammatory factors that mitigate recovery events and exacerbate neuronal cell death. It is considered possible to increase the longevity of implantable therapeutic devices in the CNS by reducing glial scarring, attenuating pro‐inflammatory secretions from immune cells (notably microglia), and promoting a regenerative environment at the injury site.

Therapeutic intervention to this dynamic environment should ideally attenuate inflammation and enhance the survival and function of endogenous cells; however, the challenge is compounded by the loss of ECM and the requirement for efficient systems to locally sustain drug delivery at therapeutic concentrations. Many neuroprotective drugs (proteins and molecules) are ineffective after systemic administration due to the BBB, formed by the brain capillary endothelium, limiting their entry into the brain,^[^ ^33^, ^34^ ^]^ or they are quickly degraded and eliminated in the circulatory system^[^ ^35^ ^]^ This problem is exacerbated for large molecular weight drugs.

Acute injuries have a complex pathophysiology, so researchers have increasingly employed a combinatorial approach, utilizing biomaterial‐based treatments, to develop a platform approach that can replace the function of the regenerative ECM for long enough to allow innate processes to take over. These materials must wholly or partially perform multiple functions including matching the structure and composition of healthy tissue, protecting degenerating neural cells, overcoming the BBB, replacing the lost neurons using endogenous/exogenous stem cells, promoting angiogenesis, and providing a conducive microenvironment for regenerated cells to survive and integrate into host neural circuits.

Understanding these key features and research targeted to address them via physical and chemical modification would allow the design and fabrication of superior biomaterials. This review identifies the pivotal physio‐chemical components for biomaterials, with a focus on self‐assembling peptide hydrogels, that enable reduction of the foreign body reaction, delivery of therapeutic agents to injury sites and modulate physiological responses.

## 2 Extracellular Matrix Mimics

In nature, the ECM performs a wide range of functional roles. It hydrates tissues, provides structural support, and gives cells a reference for their function and activity. The ECM is a porous micro‐ /and nano‐structured substrate made of various fibrillar proteins that are extensively cross‐linked with each other and intertwined with a glycosaminoglycan network. This network is resilient to tension, compression, and mechanical stress. Moreover, ECM proteins have cellular binding sites that have a pivotal role in cell adhesion, migration, proliferation, and differentiation. Therefore, the ECM represents a complex and dynamic system that modulates and influences various cellular functions at the organ and tissue level. Replicating these physiological structures can be beneficial for a scaffold's efficacy^[^ ^36^ ^]^

Biomaterial development, to fabricate a replacement ECM, has focussed on synthetically reproducing one or more of these key aspects. Severe CNS injuries result in limited spontaneous tissue repair or regeneration due to the formation of tissue lesions that are separated from adjacent viable neural tissue by dense glial scars. These scars are formed by NG2 glia, microglia, and reactive astrocytes (expressing elevated levels of glial fibrillary acidic protein (GFAP)).^[^ ^37^, ^38^ ^]^

This approach seeks to address some of the clinical shortcomings of the existing approaches; minimize systemic exposure and associated side effects; and promote brain repair and regeneration. Material scaffolds should be capable of performing multiple functions simultaneously: i) be readily and safely implantable ii) provide mechanical support to damaged cells and surrounding tissue and iii) provide sustained local release of exogenous therapeutics and anti‐inflammatory molecules.

However, as a consequence of any kind of biomaterial implantation, including metals, organics or inorganics, natural or synthetic polymers,^[^ ^39^, ^40^ ^]^ there is activation of an immune response^[^ ^41^ ^]^ After implantation, blood and interstitial fluid proteins can adsorb to the biomaterial surface, causing a host immune response^[^ ^42^ ^]^ Little is known about how such responses influence biomaterial function in vivo. Biomaterial properties, such as physical properties (topography, fiber thickness and mechanical properties), chemical properties (surface charge, wettability, surface chemistry, functional motif sequences) can be included to modify the material's biological properties and the subsequent interactions of host cells with the biomaterial.

**Figure 2:**
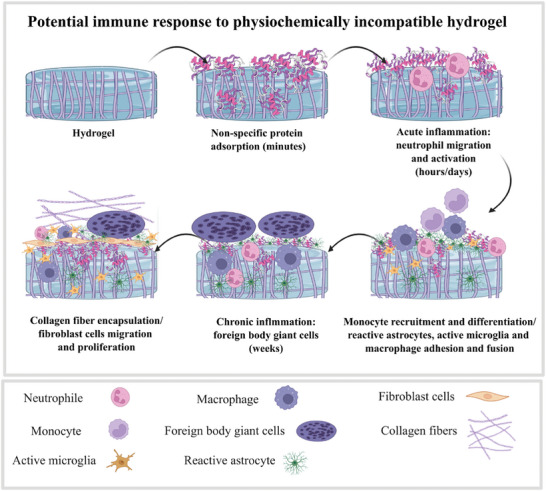
Schematic representation of the main stages involved in inflammatory responses to the non‐conducive biomaterial implantation in the CNS. Non‐specific protein adsorption on the surface of the biomaterial leading to acute inflammation, collagen encapsulation, cell migration and proliferation, chronic inflammation, monocytes recruitment and differentiation, reactive astrocyte (astrogliosis) and active microglia infiltration, and finally macrophages adhesion and fusion. Created with BioRender.com.

Foreign body reaction (FBR) is a cascade inflammatory response which gives rise to the formation of fibrosis, cellular and collagenous deposition leading to engraftment impediment or rejection^[^ ^39^, ^43^ ^]^ (**Figure** **2**). FBR as a natural protective mechanism of the host body, largely affects the performance and durability of the implanted biomaterials. On the other hand, the immune system components have a positive influence on tissue regeneration and healing. So, the ultimate result of FBR can either be chronic inflammation or wound healing, depending on the factors involved in the host response and host reactions to the biomaterial following implantation.^[^ ^44^, ^45^ ^]^

Host response to a foreign material in brain tissue is mostly carried out by the microglia^[^ ^46^ ^]^ The roles of the microglia are diverse and critical to the capacity of the nervous system to recover from injury, however the microglia can also contribute to the damaging effects of sustained inflammation. Continued presence of foreign materials results in the formation of a dense gliosis layer surrounding the material characterized by the presence of hypertrophic astrocytes to protect adjacent vulnerable neurons. It is crucial to observe the response of microglia and astrocytes to adequately assess the biocompatibility of any potential material for brain tissue application. In vivo, the degree and number of reactive microglia and astrocytes surrounding the biomaterial are often used as an indicator of immunorejection or bio‐incompatibility^[^ ^47^ ^]^ As such, the success of biomaterials are determined by an interplay between inflammation and its resolution, the latter ultimately necessary for wound healing^[^ ^48^ ^]^ Although the host immune response to an implant cannot be completely avoided, it is imperative to minimise fibrosis and inflammation at the delivery site to ensure bio‐integration and functional recovery.

To date, several attempts have been made to develop biomaterials that can regulate immune responses and consequently reduce the capsule formation.^[^ ^42^, ^49^, ^50^ ^]^ For example, efforts to attenuate the host inflammatory response to implants have resulted in the development of immune‐isolating materials to coat implant surfaces^[^ ^.51^ ^]^ Various types of zwitterions have been developed in recent years including betaine‐based zwitterions such as phosphobetaine with a phosphate group and a terminal quaternary ammonium group, sulfobetaine (SB)^[^ ^52^ ^]^ carboxybetaine (CB)^[^ ^52^ ^]^ and phosphorylcholine^[^ ^53^, ^54^, ^55^ ^]^ (a major component in the cell membrane which belongs to the phospholipid polymers class) materials, and zwitterionic amino acids/peptides. These materials have antifouling properties that result in the reduction of protein adsorption and immune response. Although zwitterionic coatings have been shown to be very potent in preventing protein adsorption, limitations around inhibiting cell adhesion^[^ ^56^ ^]^ and poor biodegradation, should not be ignored. Hence, such materials may not be suitable as scaffolds where the cell attachment to the biomaterial is important.

Therefore, the physical and chemical properties of biomaterials can be tailored to reduce the occurrence of immune responses. An artificial scaffold can be modified by controlling scaffold morphology, architecture and components, suitable topographical and biochemical cues to provide a conducive substrate for axons to penetrate the injured area in CNS tissue engineering^[^ ^57^ ^]^

### 2.1 Hydrogels as a Biomimetic ECM

Organ regeneration can be enhanced through the introduction of biomaterial implants to mimic the in vivo 3D microenvironment that allow integration with the host tissue to facilitate repair and regeneration of damaged tissues and provide physical support while preventing the inflammatory response. Hydrogels are cross‐linked hydrophilic polymer networks that hold a large amount of water, which are broadly being used in the therapeutics for ischemic diseases and many other aspects experimentally and clinically^[^ ^58^ ^]^ Due to this high‐water content, which can replicate hydrated native microenvironments and their adaptability as responsive, injectable and biodegradable, hydrogels are promising candidates for designing immunomodulatory materials.^[^ ^59^, ^60^, ^61^, ^62^, ^63^, ^64^, ^65^ ^]^ Hydrogels can be formed with different gelators including natural biological sourced materials such as acellularized tissue and ECM‐derived macromolecules (e.g., collagen, hyaluronic acid (HA), chitosan), synthetic molecules such as poly(ethylene glycol (PEG) or polyacrylamide, polymetharylamide, and synthetic supramolecular hydrogelators including self‐assembling peptides (SAP) or monosaccharides. The benefits and applications of biologically‐derived and synthetic hydrogels are reviewed in **Table** **1**.

**Table 1:** Benefits and applications of biologically derived and synthetic hydrogels in neural applications. Hydrogels made of biologically derived materials such collagen, hyaluronic acid, alginate, fibrin, chitosan, xyloglucan, and methylcellulose have been used in treatment of CNS injuries. pHPMA, pHEMA, and PEG are also the most investigated synthetic hydrogels for CNS treatment.

Hydrogel composition	Benefits	Applications	Ref
**Biologically derived hydrogels**			
Collagen	Loading and controlled‐release of nerve growth factor and bone marrow‐derived mesenchymal stem cells. Support neural cell attachment and proliferation, thus enhancing reconstruction of neural networks. Demonstrated recovery of motor function.	Traumatic brain injury	[66, 67, 68, 69]
Hyaluronic acid	Constituent of the brain ECM. Chemically and physically modified with brain regenerative factors and reactive oxygen species. Promoting the motor, learning and memory ability.	Diseases and injury in the central nervous system	[70, 71, 72, 73, 74]
Alginate	Maintaining neural cells in the undifferentiated state with pluripotent capabilities without any enzymatic treatment. Proper mechanical properties. Localized drug release.	Neural cell culture Spinal cord injury Drug delivery Cell‐based therapies	[75, 76, 77]
Fibrin	Improving the survival and migration of the transplanted cells. Loading with tumoral necrosis factor. Accelerating nerve regeneration.	Spinal cord injury Peripheral nerve regeneration	[78, 79]
Chitosan	Biologically relevant 3D scaffold for neural tissue engineering. Proper osmolarity for promoting cell survival. Loading with Metformin, thus promoting the upregulation of neural regeneration–related proteins. Loading with ferulic/succinic acid, thus increasing tissue regeneration and decreasing the inflammatory reactions.	Neural tissue engineering Stem–cell neural regeneration Brain injury	[80, 81, 82]
Xyloglucan	Thermoresponsive scaffold to assist the regeneration of the injured spinal cord.	Spinal cord injury	[83]
Methylcellulose	Functionalizing and loading with biological moieties to promote neural regeneration. Tunable mechanical properties. Thermoresponsive properties.	Central nervous system injuries	[84, 85]
**Synthetic hydrogels**			
Poly(N‐2‐(hydroxypropyl)methacrylamide) (pHPMA) Poly(hydroxyethylmethacrylate) (pHEMA)	Modified with biological moieties. Promoting ingrowth of connective tissue Nerve guidance	Parkinson's disease Nervous tissue repair Extensive spinal cord spinal cord injury	[86, 87, 88]
Polyethylene glycol (PEG)	Promoting neural stem cell migration and differentiation. Proper mechanical properties. Delivery of hydrophobic drugs for site‐specific delivery without systemic circulation.	Injuries to and diseases of the CNS Drug delivery against glioma recurrence	[89, 90, 91]

#### 2.1.1 Self‐Assembling Peptide Hydrogels Can Reproduce Features Cells Recognize

Internal architecture and mechanical properties are characteristics that must be defined when designing a hydrogel. For example, at the microscopic level: its biochemical signature, stiffness, pore size, porosity, swelling, and viscoelasticity, are all factors that will affect tissue regeneration properties at the lesion site.^[^ ^92^ ^]^ Polypeptide hydrogels formed by peptide self‐assembly are promising polymeric biomaterials owing to their sequence and structure relationship and the possibility to optimize their characteristics without significant batch to batch differences. Functionally designed SAPs have been increasingly explored as tuneable biophysical and biomechanical materials for neural tissue engineering and preparation of advanced materials for CNS regenerative medicine.^[^ ^93^, ^94^ ^]^ These SAP gels are capable of creating a 3D environment for cell migration, glial scar prevention, and axonal extension.^[^ ^95^, ^96^ ^]^ In addition, they have many inherent properties, including i) a minimal risk of carrying biological pathogens or contaminants,^[^ ^97^ ^]^ ii) a highly hydrated 3D environment to promote cell growth and migration, similar to the native ECM of host tissue;^[^ ^98^, ^99^ ^]^ iii) excellent physiological tunability, compatibility, as well as minimal cytotoxicity owing to its composition of naturally occurring amino acids;^[^ ^100^ ^]^ iv) ease of synthesis and customization with bioactive moieties, v) capacity to flow and gelate in even hard‐to‐access brain lesions and fill the cavities, regardless of their size and shape;^[^ ^95^ ^]^ vi) stable secondary structures (α‐helix, β‐sheet, or random coil)^[^ ^98^, ^101^ ^]^ and vii) long term biocompatibility and biodegradability (hydrolytic or enzymatic degradation).^[^ ^102^, ^103^ ^]^

In the case of noncovalent bonds and spontaneous organization of peptides, self‐assembly is facilitated, which leads to the formation of supramolecular structures such as nanotubes, nanospheres, nanofibers, vesicles, micelles and sheet‐like structures.^[^ ^104^ ^]^ This organization is usually triggered by changes in temperature, pH, or by addition of monovalent or divalent electrolyte ions.^[^ ^97^, ^105^, ^106^ ^]^ Typically, SAPs are composed of alternating positive and negative amino acids (or alternating hydrophobic and hydrophilic amino acids) that assemble into nanofibrous networks capable of entrapping water. These engineered biocompatible nanofibrous scaffolds can serve as a bridge for axonal regeneration or as cell/drug carriers.^[^ ^107^, ^108^ ^]^

Most reported SAP scaffolds are based on a β‐sheet motif which can form indefinite high aspect‐ratio nanofibers. Therefore, the application of such peptides has been focused on the sustained drug delivery, wound healing, modulation of angiogenesis, tissue engineering, and inflammation.^[^ ^109^, ^110^ ^]^ In the following sections, we discuss how to design desired immunomodulation by regulating the physical and chemical characteristics of hydrogels. We define “physical properties” as structural and mechanical properties including dimensionality (2D or 3D), stiffness, porosity and nanotopography; while “chemical properties” include wettability (hydrophilicity/hydrophobicity), surface charge, and molecular presentation.

## 3 Bioinstructive Physiochemical Modifications of Self‐Assembled Peptides

The hierarchical structures that underpin SAP hydrogels make them versatile and promising materials for biomedical applications. However, some essential considerations are required when designing SAP hydrogels for tissue repair in vivo; the basic small molecule subunit must enable a stable structure under physiologically relevant conditions while ensuring such structures can also provide a conducive microenvironment by mimicking physiochemical properties of the brain tissue and regulating cell behavior, while not elevating FBR upon implantation. To this end, the amino acid sequence that forms the backbone can be tuned to design unique self‐assembling peptide nanostructures with specialized physiochemical properties.^[^ ^111^ ^]^ The stiffness, topography, and/or chemistry of biomaterials have synergistic effects, so that the surface stiffness is dependent on the surface composition and topography.^[^ ^112^ ^]^ It is possible to generate SAPs capable of forming structures with higher selectivity and stability than traditional non‐biological materials to achieve optimal biological performance.

### 3.1 Mechanical Stiffness

Stiffness (quantified as storage modulus, G’) is defined as the ability of the material to resist deformation in response to an applied force.^[^ ^113^ ^]^ It should be noted that stiffness is a bulk measure; it arises as a function of the underpinning connections in the matrix that forms the hydrogel, which are determined by the SAP sequence, rate and conditions of assembly.^[^ ^114^, ^115^ ^]^ It has become apparent that nervous tissue cells are highly sensitive to these mechanical properties. Through the transmembrane adhesion receptors, cells respond to the environmental stiffness translating mechanical stimuli into biochemical responses, including cell adhesion, migration, proliferation and differentiation. This is defined as mechanotransduction. In that context, tissue stiffness plays a role in guiding or modulating cellular events, from early embryogenesis to tissue repair.^[^ ^116^ ^]^

**Figure 3:**
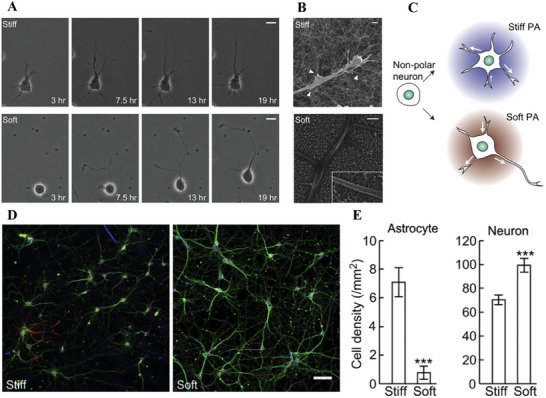
The effect of peptide amphiphile (PA) nanofiber substrate stiffness on neurite growth and astrocyte/neuron density in culture. A) Neurite growth on stiff (top) and compliant (bottom) PA substrates. Scale bar = 10 mm B) The interaction between neurites and nanofibers was visualized using scanning electron micrographs, with stiff PA nanofibers (top) attached to neurites in bundles (arrowheads) and compliant nanofibers (bottom) attached as individual fibers, scale bar = 1 mm. C) Schematic illustration of the effect of PA stiffness on motility of neurite and polarization. D) The distribution of neurons on stiff (left) and compliant (right) PA substrates was visualized with immunolabeling using β‐tubulin III (green), astrocytes (GFAP, red), and (DAPI, blue), scale bar = 50 mm. (E) Quantification of astrocytes and neurons density in hippocampal culture (number/mm^[^ ^2^ ^]^) on stiff and compliant PA substrates (***p < 0.001, *n* = 3). Reproduced with permission.^[^ ^128^ ^]^ Copyright 2013, Elsevier.

Appropriate stiffness of a biomaterial is necessary for stable network formation as a scaffold and biocompatibility. For example, stiffer biomaterials or modulus mismatch between the biomaterial surface and host tissue can cause severe FBR and reduced biocompatability.^[^ ^117^ ^]^ Implanted biomaterials' stiffness affects macrophage behavior, including their ability to phagocytose, which is linked to their polarization.^[^ ^41^ ^]^ As such, substrate stiffness must be considered in developing biomaterials as an important physical parameter to match the local biological tissue. The stiffness of nervous tissue varies depending on tissue type and location with the range of (100–1000 Pa)^[^ ^118^, ^119^ ^]^ which is similar to SAP nanofibrous scaffolds. Most neuronal and glial cells respond differently to the substrates with varied stiffness and adapt their morphology and cytoskeletal composition accordingly.^[^ ^120^, ^121^, ^122^ ^]^ It has been shown that a stiffness range exceeding 200 kPa can lead to apoptotic activity and reduced viability of neural cultures.^[^ ^123^ ^]^ Therefore, adverse reactions can be attenuated with stiffness adjustment.^[^ ^124^ ^]^ By way of example, neurons can thrive and extend neurites with multiple branches on compliant substrates better.^[^ ^122^, ^125^ ^]^ It has also been reported that the fate of differentiating stem cells is largely controlled by substrate stiffness.^[^ ^118^ ^]^ This observation was further confirmed with the propensity of neural stem cell differentiation toward neuronal cells on substrates with G’<1 kPa and glial lineage on substrates with G’>1 kPa.^[^ ^126^ ^]^ Additionally, primary astrocytes and microglia exhibited different morphology, gene/protein expression changes with substrate stiffness.^[^ ^124^ ^]^ Astrocytes on compliant gels had a spherical shape or stellate morphology with fine processes resembling their in vivo shape, while the stiffer substrate (G’ = 10 kPa) extended several processes and displayed a polygonal shape^[^ ^124^ ^]^ It has also been reported that axon length and degree of spreading vary with substrate stiffness^[^ ^127^ ^]^ In another study by Sur et al. the effect of varying gel stiffness (22.9 and 7.3 kPa) was explored on hippocampal cells^[^ ^128^ ^]^ The intensity of astrocytes on a stiffer hydrogel was reported to be 10 times higher than the softer hydrogel. Moreover, they reported 30% lower neuronal density on stiffer substrates compared to compliant self‐assembled fibers^[^ ^128^ ^]^ (**Figure** **3**).

It has been shown that substrate stiffness affect axon length and its spreading scale.^[^ ^127^ ^]^ Compliant hydrogels stimulate exploratory growth of axons,^[^ ^129^ ^]^ facilitate synaptic formation,^[^ ^129^ ^]^ and induce M2 polarization (elevated secretion of IL‐10, CD206, and Arg‐1). Cells grown on stiff substrates tend to exhibit straighter and more parallel growth of axons.^[^ ^129^ ^]^ They are also more likely to display M1 polarization with high levels of IL‐1ß, TNFα, CXCL11, and CCL20 expression and strong FBR.^[^ ^130^, ^131^ ^]^ The increase in stiffness in glial scarring post CNS damage may also explain this effect, which results in significant neural loss and cell death.^[^ ^132^, ^133^ ^]^

Tuning of SAP hydrogel biomechanics (mechanical properties) can be influenced by several factors: i) sequence charge, branching, and their position within the peptide,^[^ ^134^, ^135^ ^]^ ii) chiral amino acid arrangement, iii) peptide length and concentration,^[^ ^136^, ^137^ ^]^ iv) self‐assembly time, v) polymer chains and the conditions during gelation,^[^ ^138^ ^]^ vi) the level of hydrophobic residues, the number of entanglements^[^ ^139^ ^]^ and vii) various ions^[^ ^140^ ^]^.

One simple way to alter the mechanical properties of SAP hydrogels is by adjusting their concentration.^[^ ^141^, ^142^ ^]^ This can range from the minimum concentration needed to form supramolecular assemblies to the maximum concentration that provides aqueous stability. Changes in concentration alter SAP hydrogel self‐assembly kinetics, pore size, final nanostructures, and the density of bioactive cues tethered to the SAP backbone. The main drawback of tuning the mechanical properties via concentration is that it can influence the overall porosity of the resultant scaffold, and lead to off‐target structures that are outside the design parameters. The higher concentration of peptide results in higher bulk density of functional motifs which can influence cell binding affinity and cytokine diffusion.^[^ ^143^ ^]^

Mechanical properties can dictate degradation rate. However, following degradation the mechanical properties shift from their initial state. For example, hydrogel degradation occurring through either surface erosion (degradation that takes place at the exterior surface, while the inside of the material does not degrade until all the material immediately surrounding it has been degraded), or bulk erosion (cleavage of individual bonds within the polymer where degradation occurs throughout the whole matrix homogenously) will have an impact on the mechanical properties of the hydrogel.^[^ ^144^, ^145^ ^]^ The majority of hydrogels exhibit bulk erosion because of their high water content and permeability.^[^ ^146^ ^]^ Over time, biodegradation increases scaffold porosity and allows cell infiltration, but it adversely affects mechanical integrity. Therefore, it is a paramount need to engineer a compatible biomaterial that provides mechanical support and allows tissue growth to achieve regeneration and degrades overtime to allow growth of the new tissue and avoid adverse responses. The degradation rate of hydrogel matrices can be controlled by several factors including molecular weight, pore density, and the degree of cross‐linking.^[^ ^147^ ^]^

Ideally, scaffolds mimicking the ECM should degrade and be replaced by natural tissue. The degradation rate of the biomaterial must be precisely tuned with the cellular proliferation to achieve successful recovery. For large and irregularly shaped lesions caused by TBI, it is preferable to have long‐term degrading scaffolds (until the cells populate the lesion site and secrete their own ECM) providing structural support for the adjacent brain parenchyma, while also supporting cell differentiation. For example, hydrogels with a slow degradation rate support transplanted cell survival, ECM development, and host tissue integration.^[^ ^58^, ^64^, ^148^ ^]^ Contrary, having a fast degradation can decrease the therapeutic efficacy of an embedded drug increasing the risk of undesired side effects such as the accumulation of chemical degradation products, and higher inflammatory molecules, which in turn can encourage glial scarring and immune/foreign body response and cell death.^[^ ^145^, ^149^, ^150^ ^]^ However, immunogenic, and inflammatory response is sometimes observed in long‐term degrading, permanent, or non‐resorbable implants. As a result, for successful integration into neural networks, the chemical properties of the material must allow it to degrade slow enough for cell integration and tissue growth, while only produce biocompatible degradation products to prevent immune response.

For slow degrading hydrogels, matrix metalloproteinases (MMPs)‐cleavable peptide linkages like LIG or LRG can be inserted in the middle of peptide sequence to accelerate the degradation and subsequently facilitate cell migration.^[^ ^151^ ^]^ On the other hand, for large brain lesions caused by TBI, it may be beneficial to use a peptide matrix that degrades slowly to provide long‐term physical support to the adjacent brain parenchyma. Most commonly, amino acids and peptides are modified or conformationally altered to enhance hydrogel stability. As an example, to achieve metabolically stable peptide hydrogels, β‐amino acids with an additional carbon atom compared to α‐amino acid peptides have been developed.^[^ ^152^ ^]^ Another approach is the replacement of the synthetic D‐amino acid by natural L‐amino acid counterparts. The D‐form peptide bond resists L‐enzyme degradation and has proved to be more stable.^[^ ^153^ ^]^ Another approach to increase the stability and stiffness of the formed supramolecular hydrogels is through a technique called covalent capture.^[^ ^154^ ^]^ The covalent capture methodology is an effective approach to create highly stable supramolecular peptide‐hydrogels through non‐covalent self‐assembly following covalent stabilization to the structure. However, these injectable hydrogels designed to undergo in situ chemical cross‐linking, have limited control over gelation kinetics that can limit their utility in focally supporting implanted cells. In this regard the desirable material is one that upon injection rapidly undergoes a solution to gel (sol‐gel) transition to achieve a void filling flow regime, ensuring efficient delivery and better stability.^[^ ^155^ ^]^ Using tissue adhesive sequences can help avoid or minimize the migration due to their binding affinity to integrins found on the cell membrane. Another approach to improve material retention is the use of hydrogels with shear‐thinning properties.^[^ ^156^ ^]^ Shear‐thinning is a rheological property of SAP hydrogels that allows for injectability of the pre‐formed stable gel after subjecting the material to stress. Upon removing the stress, the material will self‐recover and return to its original structure. Moreover, shear‐thinning/self‐healing kinetics is considered an important factor in determining the suitability of the SAP hydrogels for biomedical applications. For instance, the mechanical properties of these shear‐thinning materials should allow hydrogel to flow under shear stress (the amount of applied shear should be optimal as it can affect cell survival) and at the same time recover fast to prevent sedimentation or leakage of the encapsulated cargo. This can be achieved due to the fragile noncovalent cross‐linking bonds through the intra‐ and intermolecular self‐assembly that can easily break and reform.^[^ ^157^ ^]^ SAP hydrogels with shear‐thinning and self‐healing properties can be easily delivered into even hard to access sites, fill irregular cavities, provide maximal interfacial contact with the tissue, and allow the drug to elute directly into the local microenvironment. Therefore, these shear‐thinning hydrogels are efficient as therapeutic agents/cell carriers owing to their dynamic nature. As way of example, neural stem cell (NSC) encapsulation in RADA16‐IKVAV, not only enhanced the survival of the encapsulated NSCs but also attenuated the glial astrocytes formation within the injured brain.^[^ ^158^ ^]^ In another study, the inclusion of neural progenitor cells into K_2_(QL)_6_K_2_ peptide scaffold improved neuronal differentiation, suppressed the astrocytic development of NPCs, and improved behavioral tests in an SCI model.^[^ ^159^ ^]^ These results further highlight the advantages of using shear‐thinning SAP hydrogels to protect cells from suffering membrane damage due to mechanical forces and ensure cell survival during syringe‐needle flow.

Another important factor in tissue regeneration, which is attributed to stiffness, is neovascularization. The formation of new capillaries in the tissue neighboring to the implant is believed to be capable of sustaining nutrient supply to the implant, thereby overcoming the problem caused by collagen capsule formation as a result of FBR.^[^ ^39^, ^160^ ^]^ In a study, RADA16‐SVVYGLR SAP hydrogel implantation in zebrafish resulted in sprouting angiogenesis, developmental neurogenesis, and functional recovery.^[^ ^161^ ^]^ Mechanical properties can have a significant impact on vasculature capability to penetrate the construct. For example, vascular ingrowth can occur more easily when softer materials are applied.^[^ ^162^ ^]^

### 3.2 Defining Dimensionality and Nano to Microscale Topography

Aside from a substrate's stiffness, surface architecture and topography and adhesion receptors can also affect cell response.^[^ ^163^ ^]^ Topographical features including shape, size, porosity, geometrical structure, and surface roughness can direct cellular functions, modulate immune responses, and determine the biocompatibility of the implant. The possibility of influencing a desirable immune response primarily through modulating a biomaterials surface topography is an appealing option, as does not interfere with the delicate biochemical environment and can be included in the design strategy.

In the 3D environment, the scaffold's fibers and pores must be much smaller than the cells, so that the cells can be fully embedded in the nanofibrous scaffold, similar to the native ECM.^[^ ^164^ ^]^ In the case of microfibers, cells follow the curvature imposed by the microfibers topography. These platforms serve as 2D scaffolds with curvature as they contain porosities (≈100 µm) greater than the average cell diameters (≈10 µm). In this context, SAP hydrogels with their interwoven nanofibers ≈10–20 µm in diameter with porosities between 5 and 200 µm could provide a true 3D environment and are the most suitable for nerve growth.^[^ ^165^, ^166^, ^167^ ^]^

Scaffold orientation, at the micro/nano scale level, has been shown to affect the immune responses with the least amount of monocyte adhesion on aligned‐oriented scaffolds compared to randomly oriented fibers on electrospun polycaprolactone scaffolds.^[^ ^168^ ^]^ It has been reported that 4 weeks post‐transplantation, aligned fiber scaffolds were surrounded by significantly thinner fibrous capsule in vivo. Aligned fibers demonstrated higher cell infiltration within the scaffold while randomly aligned fibers exhibited accumulated cells on the surface. Aligned topography is found to be among the most effective in neural tissue regeneration, where cell infiltration into the scaffold matrix is very important.^[^ ^123^, ^163^, ^169^, ^170^ ^]^

**Figure 4:**
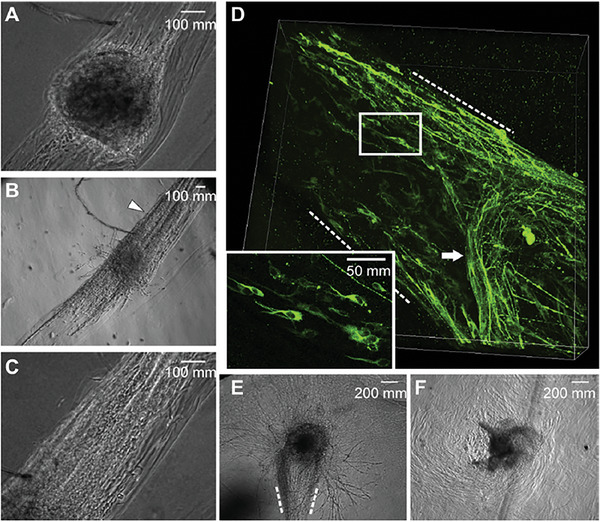
Scaffold alignment guided cell migration. Aligned scaffolds made of IKVAV PA were used to encapsulate dorsal root ganglia (DRGs) being incorporated in a collagen gel. Bright field representative images of DRG 1 day A) and 6 days B) post culture. C) A magnified representation of the area pointed out by the arrowhead in image (B). D) A 3D representation of DRG stained with β‐III‐tubulin after 6 days of culture inside an aligned scaffold. The scaffold boundary is indicated by dotted lines, and a magnified view of a section from the region marked by the rectangle is shown. DRG cells migration when E) located at position at the extremity of an aligned scaffold (as indicated by dotted lines) or when F) embedded within an unaligned PA scaffold. Reproduced with permission.^[^ ^177^ ^]^ Copyright 2014, Elsevier.

Human neural stem cells (hNSCs),^[^ ^171^, ^172^, ^173^ ^]^ human embryonic stem cells (hESCs),^[^ ^174^ ^]^ and human induced pluripotent stem cells (hiPSCs)^[^ ^175^ ^]^ can differentiate toward the neuronal lineage when exposed to aligned microscale patterns, aligned ridge pattern, and aligned microgrooves. This cellular response toward aligned fibers is highly desirable for neural regeneration. In an attempt to produce aligned peptide nanofibers, an aqueous peptide amphiphile (PA) was injected into CaCl_2_‐containing physiological saline to form noodle‐like hydrogel.^[^ ^176^ ^]^ This aligned PA was later functionalized with the bioactive epitopes IKVAV (isoleucine‐lysine‐valine‐alanine‐valine) and RGDS (arginine‐glycine‐aspartate‐serine) and was shown to promote aligned neurite outgrowth in P19 (embryonal carcinoma cell) mouse neurons.^[^ ^177^ ^]^ Moreover, dorsal root ganglion (DRG) cells showed extensive migration in the scaffold, with their direction guided by the fiber alignment^[^ ^177^ ^]^ (**Figure** **4**).

In another study, the PA solution (RGDS‐PA, IKVAV‐PA) was loaded into the porous poly(lactic‐coglycolic acid) PLGA tubes. Then PA solution passed through a 40‐mm mesh screen under shear flow into a 20 mm CaCl_2_ bath. The resultant nanofibers were aligned in the direction of fluid flow in order to spatially guide nervous fiber regeneration.^[^ ^178^ ^]^ These results demonstrate the potential of peptide materials to provide directional cues to neurons and direct neurite growth.

### 3.3 Scaffold Porosity

In natural tissues, cells situated in a porous ECM, which occupies ≈20% of brain tissue. The ECM provides confined spaces with specific geometries that facilitate capture of nutrients, diffusion of waste products and cell–cell communication.^[^ ^179^ ^]^ Hydrogels with interconnected porosity can facilitate these throughout the 3D structure,^[^ ^65^, ^180^ ^]^ and regulate immune cell responses, by facilitating cell migration and infiltration inside hydrogels while maintaining physical stability.^[^ ^181^ ^]^ Moreover, hydrogel porosity effects the progression of FBR by regulating the release and retention of bioactive molecules, directing desired immunomodulation.^[^ ^182^ ^]^ It has been reported that porous hydrogels induced macrophage infiltration and enhanced M2 polarization as well as blood vessel formation leading to the acceleration of integration with the surrounding tissue.^[^ ^162^, ^183^ ^]^ In contrast, nutrients and oxygen within media cannot be efficiently permeated through the entire structure in hydrogels with very small pore size, potentially leading to a necrotic region.^[^ ^162^, ^182^, ^183^ ^]^ Therefore, by emulating the porosity of native tissues, desired immunomodulation can be obtained.^[^ ^64^, ^162^ ^]^ In neural applications, the pore interconnectivity is critical for neurite growth, with a desirable porosity of 90% and a suitable pore size ranging from 10 to 100 µm.^[^ ^184^, ^185^, ^186^ ^]^ Molecular design, self‐assembly molecule concentration, electrostatic capping, and the environment in which self‐assembly occurs can all affect SAP nanotopography.^[^ ^187^, ^188^ ^]^

### 3.4 Defining Surface Charge

It is increasingly evident that the surface charge density of implants can influence the quantity and conformation of proteins absorbed, which in turn affects the immune response^[^ ^189^ ^]^ Hydrogels can be designed to display different surface charges by modifying functional groups. SAP hydrogels with different charge can be achieved by regulating the ratio of alginate (R) and/or lysine (K) (positively charged) and aspartate (D) and/or glutamate (E) (negatively charged). It has been demonstrated that a net peptide charge of ±2 is optimal for the formation of self‐supporting hydrogels.^[^ ^148^ ^]^ For example, a study using multidomain peptide (MDP) hydrogels reported that MDP K2(SL)6K2 and R2(SL)6R2 peptides a high degree of collagen deposition and multiple immune cell infiltration observed, implying pronounced FBR provoked by positively charged peptides.^[^ ^190^ ^]^ In contrast, negatively charged MDP E2(SL)6E2 and D2(SL)6D2 were infiltrated by tissue‐resident macrophage to a limited extent and revealed no collagen deposition, suggesting a low level of FBR.^[^ ^190^ ^]^

**Figure 5:**
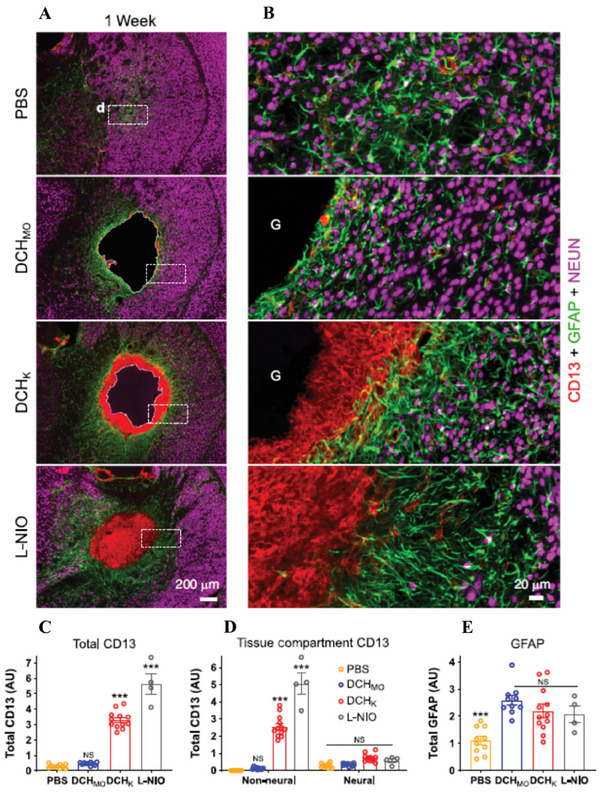
DCH hydrogel evoked different FBRs. A,B) Representative images of stromal and inflammatory cells visualized with CD13, GFAP, and NeuN 1 week after injections of PBS (to induce mild CNS wound responses), hydrogels, or L‐NIO (to induce severe CNS wound responses). C–E) Quantification data of total cells positive for (C) CD13 in CP, (D) CD13 in either non‐neural/ or neural tissue compartments, and (E) GFAP in CP. All graphs are mean ± s.e.m (*n* = 10, 10, 12, and 4 mice per group for PBS, DCH_MO_, DCH_K_, and L‐NIO, respectively, ***P < 0.0001). Adapted with permission.^[^ ^191^ ^]^ Copyright 2018, ACS.

In another report, diblock copolypeptide hydrogels (DCH), with different surface charges, were injected into the caudate putamen (CP) of a mouse forebrain. It was demonstrated that at the interface of DCH with host tissue lysine‐based (DCH_k_) cationic hydrogel materials exhibited a greater loss of neural tissue which resulted in a larger lesion and greater loss of viable neuropil.^[^ ^191^ ^]^ They also demonstrated that different hydrogels, such as DCH_k_ and methionine sulfoxide‐based DCHMO, exhibited varying intensity and cellular phenotype of the blood‐borne inflammatory response characterized by CD13 positive cells. Cationic and anionic interfaces exhibited a significantly higher level of CD13 compared to PBS and non‐ionic DCH_MO_ (**Figure** **5**).^[^ ^191^ ^]^ Thus, the severity of FBR is influenced by surface charge at the material–host interface, which can affect the degree of protective fibrotic and astrocyte barrier formation. The astroglial border serves to isolate the materials from adjacent neural tissue, which may lead to a reduction in their function.

In addition, positively charged hydrogels exhibited a higher degree of macrophage infiltration than negatively charged ones.^[^ ^191^ ^]^ This increased infiltration could be associated with enhanced cell spreading and fusion of macrophages, which may be linked to electrostatic interactions between the positively charged hydrogel surface and negatively charged cellular plasma membrane (head group of phosphatidylserine and phosphatidylinositol), as well as carbohydrate portions of glycolipids and glycoproteins.

### 3.5 Surface Functionalization Through Adding ECM‐Mimic Sequence

Cell adhesion, migration, proliferation, and differentiation in tissues are influenced by physical stimuli and inductive chemical signals. Therefore, controlling chemical signaling provides hydrogels with a significant advantage to shape the immune microenvironment. Implanting biomaterials with adhesive peptides that mimic the native ECM have yielded promising results. To increase the materials' interaction with cells, and ultimately trigger post‐injury inflammatory responses and FBR, generating a layer of biomimetic and bioactive molecules may be of benefit. To interact with a protein receptor, bioactive peptide epitopes can also be present on the surface of nanofibers. In this context, the degree of internal order in self‐assembled nanostructures is important as it controls their bioactivity and physical properties. For example, PAs with a branched bioactive sequence/functional motif were shown to form nanofibers with a low degree of internal order which provides a concentrated display of peptide epitope on the fibril surface.

Structural mimics are developed using peptide sequences that are epitopes of bioactive sites where recognition sites of the mimic are defined by both the amino acid sequence and 3D conformation. In order for the peptide to bind to the corresponding integrin receptor, its conformation is crucial. For this reason, biomaterial surfaces are often functionalized with longer peptide chains^[^ ^192^ ^]^ and cyclic peptides^[^ ^193^ ^]^ to optimize cellular interaction.

As a means of replicating developmental steps and promoting regeneration in the adult brain, ECM molecules have been used to biofunctionalize synthetic scaffolds. One way to provide cell attachment to the material is the incorporation of peptides that display a specific binding sequence.^[^ ^194^ ^]^ Through binding of cell adhesive molecules/functional motifs, interactions between the implanted hydrogel and endogenous neural cells induce many different reparative and anti‐inflammatory cellular pathways. To initiate these cell pathways, scaffolds have often been modified with ligands that target integrin receptors on the cell surface. Laminin is a pivotal substrate along which nerve axons will grow and incorporate into scaffolds to enhance cell adhesion and migration.^[^ ^195^ ^]^ Natural ECM proteins such as fibronectin and laminin are the most important integrin proteins providing great cellular attachment and can be mimicked by short peptide motifs sequences namely RGD,^[^ ^196^ ^]^ IKVAV,^[^ ^197^ ^]^ and tyrosine‐isoleucine‐glycine‐serine‐arginine (YIGSR). RGD is highly effective at promoting cell attachment,^[^ ^198^ ^]^ YIGSR sequence possess capability to promote not only neurite outgrowth but also facilitates the cell binding^[^ ^199^ ^]^ and the IKVAV peptide facilitates the neurite extension.^[^ ^64^, ^200^ ^]^ These sequences, often called ultrashort peptide sequences, by definition do not contain more than seven amino acids. The presence of N‐terminal protecting groups have been found to play a critical role in ultrashort peptide self‐assembly through manipulation of the pKa and hydrophobic *π*–*π* stacking of the fluorenyl groups.^[^ ^201^, ^202^, ^203^ ^]^ These amino acids with a 9‐fluorenylmethoxycarvonyl (Fmoc) typically used for N‐terminus protection in solid phase synthesis, form fibril nanostructures. Fmoc‐protected peptides are promising for the fabrication of supramolecular soft biomaterials. Fmoc derivatives of different libraries of ultrashort peptides have found significant success, and are reported to further reinforce the role of *π*–*π* stacking interaction in driving the self‐assembly, i.e., Fmoc‐LD, Fmoc‐RGD, Fmoc‐FG, Fmoc‐RGDF, Fmoc‐FRGD, Fmoc‐AD, Fmoc‐FF, and Fmoc‐ID.^[^ ^204^, ^205^, ^206^ ^]^ Moreover, LIVAGD, ILVAGK, LIVAGK, and AIVAGD hexapeptides as well as IVD, VIE, MYD tripeptide are also reported to form stable hydrogels.^[^ ^207^, ^208^ ^]^ Some other SAP hydrogels used for neural repair applications are summarized in **Table** **2**.

**Table 2:** Examples of SAP hydrogels used for CNS repair applications.

Self‐assembling peptide (SAP) hydrogel sequence	Neuroactive sequence	Outcome	Ref
[(SL)6‐E‐G‐KKDGDGDFAIDAPE] (SL)6	KKDGDGDFAIDAPE (called CMX‐9236)/ Neuroprotective extracellular glycoprotein	Enhanced neuronal survival both in vitro and in vivo Protected cortical neurons post‐TBI and reduced atrophy	[209]
K(SL)3RG(SL)3K –G–KLTWQELYQLKYKGI	LRG, KLTWQELYQLKYKGI/ Angiogenic functional motif	Enhanced recovery from traumatic brain injury	[210]
RADA‐16‐GGSIKVAV RADA16‐IKVAV	IKVAV/ Laminin derived integrin binding motif	Enhanced survival of encapsulated NSCs Reduced glial scar formation	[158, 211]
RADA16‐YIGSR RADA16‐GGYIGSR	YIGSR /laminin derived Epitope	Promoted neural differentiation and proliferation of transplanted NSCs in Alzheimer's disease Increased neuronal differentiation and restoration of memory/learning function Rescued synaptic function, Decreased pro‐inflammatory factors in Alzheimer's mice models,	[212]
RADA16‐SVVYGLR	SVVYGLR/Angiogenic functional motif	Facilitated angiogenesis, developmental neurogenesis, and functional recovery in a brain injury model in zebrafish	[161]
RADA‐I‐GPRGDSGYRGDS	PRGDSGYRGDS/ Angiogenic functional motif	Improved angiogenesis	[213]
KSLSLSLRGSLSLSLKGKLTWQELYQLKYKGI	KLTWQELYQLKYKGI/ Angiogenic functional motif	Enhanced tissue regeneration in ischemic tissue disease	[110]
Ac‐(RADA)4‐GG‐IKVAV‐GG‐RGIDKRHWNSQ‐NH2	IKVAV/laminim functional motif and RGI (RGIDKRHWNSQ)/BDNF mimetic	Improved axonal regeneration Enhanced re‐myelination and motor functional recovery	[164]
Palmitoyl‐ NAPVSIPQKKK (PA‐NK)	NAPVSIPQKKK/ microtubule stabilising peptide	Promoted neurite outgrowth in mouse neuroblastoma cell line Enhanced neuroprotection against anti‐NGF toxicity in PC12 cells	[214]
Palmitoyl‐VVAAEEE‐ADEGVFDNFVLK‐CONH2	VFDNFVLK/ Tenascin‐C derived motif	Increased neuroblast cells (24‐fold) compared to control at 7 days post‐implantation in the ventral horn.	[215]
Lauryl‐VFDNFVLKK‐CONH2, Lauryl‐VVAGKK‐CONH2 and Lauryl‐VVAGEE‐CONH2	VFDNFVLK/ Tenascin‐C derived motif	Enhanced neural cell adhesion	[216]
Ac‐FAQRVPP‐GGG‐(LDLK)3‐CONH2		Enhanced neuronal and oligodendroglial differentiation of murine NSCs and human NSCs Improved locomotor recovery following SCI without altering the physiological inflammatory response Enhanced nervous tissue repair	[217]
K2(OL)6K2 (QL6)		Significantly reduced post‐traumatic apoptosis and the formation of glial scarring Attenuated inflammation Improved axonal conduction and tissue preservation in SCI	[218]
PPFLMLLKGSTR tethered to hyaluronic acid	PPFLMLLKGSTR/ laminin‐derived adhesive peptide	Improved cell survival and spreading of MSCs not only in the scaffold but also in the lesion site Promoted recovery from spinal cord transection Enhanced the survival of MSCs in the lesion site Demonstrated ability to bridging the gap in lesion with surrounding neural tissues (10 days after implantation)	[219]
betaVhex (KVKEVFFVKEVFFVKEVY) embedded with carbon nanotubes		Accelerated signal transmission from neurons (3‐fold higher) in betaVhex hydrogel+0.02% betaVhex/CNTs compared to the hydrogel alone during seizures in the epidural tissue	[220]

Among the short peptide motifs, IKVAV, the major protein in laminin, has become the epitome of CNS tissue engineering scaffolds as it increases the viability and maturation of neurons by binding to the β1‐integrin subunit.^[^ ^221^ ^]^ Functional epitopes within the laminin and fibronectin proteins were used to synthesize SAP hydrogels with physiologically bioactive sequences.^[^ ^222^ ^]^ Fmoc‐IKVAV, Fmoc‐DYIGSRF, and Fmoc‐FRGDF were shown to undergo optimal self‐assembly under mild, physiological conditions (pH = 7.4, 37 °C) and without the use of strong organic solvents.^[^ ^223^ ^]^ The different building blocks result in different physiochemical properties which indicate the ability of the SAP hydrogels to be tuned with the change of building blocks. Additionally, these Fmoc‐SAPs were utilised as a delivery vehicle for cell transplantation. Cortical neural progenitor cells transplanted into the mouse brain exhibited a limited FBR, effective functionalization and low cytotoxicity 28 days post‐transplantation. The results of the study further highlight the suitability of Fmoc‐SAPs for improved neural tissue repair.^[^ ^223^ ^]^

Some peptide sequences known as neural cell adhesion molecule (NCAM) peptides have shown to enhance neuritogenesis. For instance, the EVYVVAENQQGKSKA (FGL) sequence has been shown promising results to improve neuritogenesis and synaptogenesis in hippocampal neurons (in vitro).^[^ ^224^, ^225^ ^]^ Furthermore, it has been demonstrated that the aforementioned FGL peptide can reduce glia activity in aged hippocampus for both astrocyte and microglia populations.^[^ ^226^ ^]^

The concentration of bioactive sequence and its position are as important as its presence. Any over‐ or under‐expression of ECM proteins can cause serious medical implications in biological systems where their concentration is tightly regulated. As such, it is essential to optimize the concentration of bioactive motifs on the designed scaffold. The co‐assembly method is used widely to prepare different concentrations of bioactive density on the surface of SAP. To modify the bioactive sequence concentration, different volumetric ratios of (RADA)_4_/(RADA)_4_‐IKVAV were studied.^[^ ^227^ ^]^ Then the biocompatibility of the different ratio in brain tissues was examined using glial response as an important hallmark of the local host response. In the case of (RADA)_4_/(RADA)_4_‐IKVAV, different mixtures showed biocompatibility toward primary microglia cells and brain tissue.^[^ ^227^ ^]^ Laminin‐derived IKVAV peptide was chosen to promote beneficial cell interaction and attenuate glial responses.^[^ ^227^ ^]^

Additionally, RGD and IKVAV peptides were examined to determine the effects of pH and functional motifs containing these peptides. SAP hydrogels were designed by directly conjugating short bioactive peptide motifs to RADA16‐I (‐DIKVAV/‐ RGD). DIKVAV and RGD were specially designed to have opposite net charges at neutral pH. They showed that the resultant nanofiber hydrogel decorated with functional motifs (‐DIKVAV/‐ RGD) offered a more conducive environment for nerve regeneration than the RADA16 alone. The permissive environment resulted in a higher cell survival rate in (‐DIKVAV/‐RGD) hydrogels, whereas those embedded within the RADA 16‐I hydrogel survived poorly because of the low pH of the RADA16 which damages cells and host tissues upon direct exposure^[^ ^216^ ^]^

### 3.6 Wettability (Hydrophobicity/Hydrophilicity)

Surface wettability influences adsorption, denaturation, and subsequent responses of adsorbed proteins. Cell adhesion increases as the wettability/ or hydrophilicity of the scaffold surface increased.^[^ ^228^, ^229^ ^]^ It is possible to fine‐tune the hydrophobicity of SAP hydrogels through the choice of building blocks (either a self‐assembling backbone or a bioactive motif). Adding more hydrophobic residues increases the number of hydrophobic interactions, leading to the formation of stiffer scaffolds.^[^ ^217^, ^230^, ^231^ ^]^ For example, inclusion of residues, such as Ala, Val, Ile, Leu, Tyr, Phe, and Trp can substantially affect the mechanical properties of the resultant scaffold and the speed of the self‐assembly.

Increasing the hydrogel hydrophobicity improves the production of M1 and M2 cytokines, along with up‐regulation of M1 cytokine secretion, suggesting a classical shift in the phenotype of macrophages with hydrogel hydrophobicity. Moreover, the phenotypic fate and function of macrophages can also be tailored by gel wettability. For example, it has been revealed that cells on hydrophilic surfaces exhibit lower phagocytosis rates, demonstrating the importance of hydrophobicity in effective phagocytosis.^[^ ^232^ ^]^

## 4 Bioactive Molecule Delivery

It is possible for oxidative stress and neuroinflammation to persist following a TBI based on secondary injury mechanisms. Therefore, several studies on drug delivery with hydrogels in TBI use antioxidant or anti‐inflammatory drugs.^[^ ^233^, ^234^ ^]^ Moreover, many proteins, including neurotrophins and other growth factors, and also anti‐inflammatory drugs are being tested for their potential therapeutics and ability to promote brain protection, repair and regeneration.^[^ ^233^, ^234^ ^]^ Anti‐inflammatory drugs might not directly induce brain repair, but they may minimize the gliotic response of the brain to trauma and facilitate neuronal or axonal regeneration.

Neurotrophic factors are one of the key mediators of neural plasticity and functional recovery. However, despite well recognised therapeutic potency of neurotrophic factors (NTFs) (e.g., nerve growth factor (NGF), brain‐derived neurotrophic factor (BDNF), neurotrophin‐3 (NT‐3), glial cell line‐derived neurotrophic factor (GDNF), ciliary neurotrophic fact (CNTF)), efficient and targeted delivery remains a challenge,^[^ ^13^ ^]^ and issues associated with their delivery, particularly insufficient protein dose release at a target region often result in therapeutic performance below those required for clinical applications. Apart from NTFs,^[^ ^13^ ^]^ other molecules such as anti‐inflammatory drugs are also important as they are involved in minimising the gliotic reaction of the brain to trauma and facilitating neuronal and/or axonal regrowth. Brain drug delivery can be conducted through oral and transnasal administration, intravenous injection, implantation, etc.^[^ ^52^ ^]^ Among them, systemic oral and intravenous administrations, are the most deployed strategies, due to their ease of delivery. It has been reported that NTFs demonstrated fast degradation in vivo, a very short biological half‐life in circulation and poor permeability across biological obstacless.^[^ ^235^, ^236^ ^]^ Further, issues such as unclear therapeutic windows, protein instability, and the challenge of controlling release kinetics must be address. Optimum release kinetics of NTFs need to be compatible with axonal renovation and growth.^[^ ^237^ ^]^ Recently, efforts to increase the bioavailability of drugs delivered into brain, resulted in the development of various drug carriers to regulate physiochemical and biological activity of drugs, minimize the side effects, improve stability, and control drug release.^[^ ^13^, ^238^ ^]^

### 4.1 SAP Hydrogels for Controlled Drug Delivery

In contrast to vascular administration, injectable SAP hydrogels can bypass the BBB and contact the injured area directly. SAP hydrogels differ in architecture, function, and mesh size (ranges from a few to hundred nanometres) which is similar in size to most small molecular drugs and therapeutic proteins. The parameters dictate how these hydrogels are used to deliver sustained molecular release at a rate tailored to the tissue's physiological requirements. These SAP hydrogel‐mediated immune modulation can provide spatiotemporal control over the release of various therapeutic agents including small‐molecule drugs, neurotrophic growth factors, anti‐inflammatory drugs, or other macromolecular payloads to reduce FBR, improve drug availability, and have broad application prospects in the treatment of TBI.^[^ ^233^, ^234^, ^239^ ^]^ Furthermore, for optimal therapeutic efficacy, an ideal delivery system should be capable of accommodating more than one drug.^[^ ^240^ ^]^ Moreover, as they are typically formed in aqueous solutions, exposure to strong organic solvents does not lead to denaturation or aggregation of the drugs. The shrink‐swell mechanism does not affect protein release or structure because peptide hydrogels have minimal shrink‐swell capacities when they form.^[^ ^241^ ^]^

As noted previously, inflammatory cells have a regulatory role in neuroplasticity, so the complete ablation of the immune population is often detrimental.^[^ ^242^ ^]^ Therefore, in therapeutic approaches targeting post‐traumatic neuroinflammation, the damage and recovery time must be considered to avoid broad suppression of microglia while boosting the correct microglial phenotype (M2) at the right time to promote endogenous repair pathways and maximal remodelling following TBI.^[^ ^243^ ^]^ According to this study, dexamethasone loaded in the PCL electrospun nanofiber significantly reduced the FBR compared to the burst release of dexamethasone.^[^ ^244^ ^]^ These results indicated that when anti‐inflammatory drugs depleted from the coatings, the body recognized the implant as foreign material and produced a delayed chronic inflammatory response.^[^ ^245^ ^]^ Several studies have demonstrated the sustained delivery of the incorporation/encapsulation of therapeutic molecules (e.g., anti‐inflammatory drugs such as naproxen, ketoprofen, fucoidan, osteopondin, and dexamethasone) via co‐assembly or mixing within the hydrogel construct without degrading the structure to alleviate inflammatory reactions and suppress FBR in nerve interfaces.^[^ ^246^, ^247^, ^248^, ^249^ ^]^ In one study, SAP hydrogel was used as a platform for anti‐inflammatory macromolecule fucoidan payload delivery. This hydrogel markedly reduced glial scar formation compared with untreated control group. Moreover, the SAP hydrogel filled the cystic cavity as a result of trauma and provided physical support for the adjacent brain tissue, avoiding further damage caused by the collapse of the injured area. On the other hand, fucoidan presence reduced reactive astrocytes production.^[^ ^250^ ^]^

SAP hydrogel drug delivery can be designed to immobilize therapeutic molecules through physical or covalent bonds.^[^ ^251^, ^252^ ^]^ The mesh size dictates how drugs diffuse inside the hydrogel network.^[^ ^238^ ^]^ For instance, small drugs relative to the mesh size diffuse rapidly through the hydrogel and show short release duration compared to drugs with comparably the same size of the hydrogels’ mesh size. A decrease in release rate was associated with an increase in peptide hydrogel density (resulting in smaller mesh size) and an increase in protein drugs size. In addition, drugs larger than the hydrogel mesh size immobilised inside the network and release occurs through network degradation, swelling, or deformation.^[^ ^253^ ^]^ For example, in a study carried by Koutsopoulos et al. 4 different proteins (lysozyme, trypsin inhibitor, BSA, and IgG) with different physicochemical properties (pI 4.6, 11.4; molecular mass, 14.3–150 kDa) were physically entrapped in Ac‐(RADA)_4_‐CONH_2_ peptide hydrogel to investigate their release profile. Release kinetic analysis indicated that the hydrogel could retain the IgG, the larger protein, for up to 60 h, while the release of the lysozyme, the relatively smaller protein, reached the plateau after 30 h at physiological pH0.^[^ ^254^ ^]^

Additionally, SAP hydrogels are capable of delivering molecules of varying polarities due to the presence of a variety of amino acids in the design of SAP hydrogels (charged, hydrophobic, and hydrophilic amino acids). In this context, hydrophobic molecules can be encapsulated within the fibers and hydrophilic molecules are embedded external to the fibers. Prolonged drug release was observed in the hydrophobic molecule embedded within the fibers compared to hydrophilic drugs.^[^ ^255^ ^]^

Besides the protein size, the chemical properties of the protein such as electrostatic interactions, hydrophobicity, and the presence of aromatic residues can influence release kinetics.^[^ ^256^ ^]^ For instance, a study by Branco et al., was conducted to investigate the effect of differently charged molecule release from self‐assembling β‐hairpin peptide hydrogels.^[^ ^257^ ^]^ The results indicated the faster release of positively charged protein than negatively charged dextran, indicating the electrostatic interactions between the loaded molecule and hydrogel as one of the contributing factors to the release.^[^ ^257^ ^]^

Peptides can provide an appropriately biomimetic scaffold substrate and the capacity to stabilize and deliver multiple native form NTFs (e.g., GDNF and BDNF).^[^ ^223^, ^252^, ^258^, ^259^ ^]^ For example, in a study the Fmoc‐DDIKVAV hydrogel has shown the potential to prolong the delivery of BDNF for 6 weeks.^[^ ^252^ ^]^ The increase in BDNF lifespan of over 40‐fold compared to its availability in PBS alone demonstrates the capacity of the SAP hydrogels to deliver NTFs in a time‐controlled manner. In addition, as electrostatic interactions can delay release of proteins from a β‐peptide system,^[^ ^260^ ^]^ BDNF was chemically modified with chitosan. This modification resulted in more dynamic release profiles without altering the properties of SAP hydrogels^[^ ^252^ ^]^ In other study, amphiphilic diblock copolypeptide hydrogels (DCH) was used to deliver NGF neurotrophic factor to the mice forebrain. They reported that K_180_ L_20_ and E_180_ L_20_ provided sustained delivery of NGF bioactivity, which helped in maintaining the hypertrophy of local forebrain cholinergic neurons for a minimum of 4 weeks.^[^ ^261^ ^]^ The release profile of encapsulated NTFs can be fine‐tuned by altering the peptide concentration. also related to the concentration of peptide. For example, it has been reported that NGF/BDNF release was inversely related to the concentration of MAX8, a synthetic β‐hairpin peptide contains two arms of alternating lysines and valines surrounding a four‐residue sequence V^D^PPT. Moreover, the bioactivity of NTF after release from hydrogel was confirmed through the development of neurite‐like extensions of the rat adrenal phaeochromocytoma PC12 cell line in response to NGF released from MAX8.^[^ ^262^ ^]^

As mentioned earlier, functional peptide motif could mimic the biological activity of growth factors.^[^ ^263^ ^]^ Peptides are smaller than full‐length growth factors, which makes them easier to synthesize and cheaper. Additionally, peptides can interact specifically with specific receptors, which helps avoid side effects due to their small size and structural stability. This makes peptides a safer alternative to growth factors.^[^ ^264^ ^]^ Some classes of peptides have also shown to have immunomodulatory properties.^[^ ^265^ ^]^ For example, innate defence regulator (IDR) peptides are a class of peptides which have shown ability to modulate immune response. IDR‐1018 is a peptide with (VRLIVAVRIWRRNH_2_) sequence which has been shown to enhance the anti‐inflammatory responses while maintaining the key pro‐inflammatory processes important for fighting off infection.^[^ ^265^ ^]^ Another example is KHIFSDDSSE, an NCAM peptide which has been shown to interact with glia cells and limit astrocyte proliferation.^[^ ^266^, ^267^ ^]^ In a study, hyaluronic acid (HA) immobilised with an angiogenic growth factor VEGF peptide motif KLT (KLTWQELYQLKYKGI) was used to promote angiogenesis for brain tissue engineering.^[^ ^268^ ^]^ It was found that the HA‐KLT efficiently inhibited the glial scar formation at the injury sites and formed a permissive interface with the host tissues at 4 weeks post implantation. In addition, the HA‐KLT hydrogel enhanced the formation of blood vessels angiogenesis in vivo. These results, suggesting that HA‐KLT hydrogel has the potential to repair brain defects by promoting angiogenesis and inhibiting the formation of deleterious glial‐derived scar tissue.^[^ ^268^ ^]^

### 4.2 Shuttle Peptides and Nanoparticles Decorated/Conjugated with Peptides Drug Delivery Systems

A vast range of nanomaterial‐based drug‐delivery strategies have been widely developed with the purpose of facilitating drug delivery to the brain.^[^ ^269^ ^]^ In the CNS, the BBB is a critical structure with delicate characteristics that plays a vital role in protecting the brain from harmful attacks of pathogens and toxins via the blood circulation. However, the inability of drugs to cross the BBB because of their size, renders most systemically administered therapies ineffective.^[^ ^270^, ^271^ ^]^ It has been reported that ≈98% of small molecular weight drugs and almost all of large molecular weight (macromolecules) peptides/proteins fail to, or sub‐optimally, cross the BBB.^[^ ^272^ ^]^ As such, reaching therapeutic concentration at the injury site requires either local delivery or high systemic doses, while the latter leads to the systemic cytotoxicity and off‐target distribution in unexpected tissues.

Nanomaterials may need to be modified to enhance their biological performance or to mitigate their negative effects. This can be achieved by incorporating coatings from larger, bulk biomaterials. Hydrophilic and biocompatible polymers such as polyethylene glycol (PEG),^[^ ^273^ ^]^ dextran, and polyvinyl alcohol (PVA)^[^ ^274^ ^]^ are commonly used for this purpose. The attachment of these hydrophilic polymers to the nanoparticles (NPs) surface significantly was shown to diminish the binding of plasma protein, particularly opsonins, which in turn leads to prolong the circulation time by escaping from phagocytes in the reticuloendothelial system (RES).^[^ ^275^ ^]^ More recently, peptides also played a significant role in drug delivery systems to the CNS either as shuttle peptides alone or as NPs/liposomes decorations.

Shuttle peptides are molecular vectors capable of employing active, contains transport modulated by using transporters and receptors as well as adsorptive‐mediated transport, or passive, classified as either of paracellular diffusion along with transcellular passive diffusion through the lipid membrane, mechanisms, to improve transport of various compounds to the brain while also circumvent the BBB. However, thus far even with these technologies there still remain many shortcomings associated with systemic delivery using nanotechnology‐based approaches. For more information about these materials to cross BBB the interested reader is referred to the following excellent review on shuttle peptides/NP decorated peptide‐based drug delivery systems to CNS.^[^ ^276^, ^277^ ^]^ Some peptide functionalized NPs and shuttle peptides as drug delivery systems into the CNS are reviewed in **Table** **3**.

**Table 3:** Peptides as drug delivery systems into the CNS to cross BBB. Decorating NPs with several peptide sequences enhancing the drug delivery capability through BBB.

Material	Drugs delivered	Application	Route of administration	Outcome	Ref
PEG–PLA‐penetratin (RQIKIWFQNRRMKWKK)	Coumarin‐6	CNS disorders	Tail vein injection	In vivo administration resulted in significant brain uptake with lower deposition in non‐target tissues	[278]
RGERPPR‐functionalized doxorubicinliposomes (RGE‐LS/DOX)	Liposomal doxorubicin (anticancer drug)	Glioblastoma therapy	Intravenous	Tumour‐penetrating peptide functionalization is an efficient approach to boost the anti‐glioblastoma impact of doxorubicin liposomes.	[279]
Liposome encapsulated with H102 peptide (a β‐sheet breaker)	H102	Treatment of AD	Intranasal	H102 could efficiently, stably, and safely penetrate into the brain to treat AD.	[280]
Bifunctionalization of liposomes with phosphatidic acid and a modified ApoE‐derived peptide (mApoE‐PA‐LIP)	‐	AD treatment	Intravenous	mApoE‐PA‐LIP is potential nanodevice to be applied in vivo for AD treatment.	[281]
Rivastigmine liposomes (Lp) and cell‐penetrating peptide (CPP) modified liposomes (CPP‐Lp)	Rivastigmine	CNS region most affected by AD	Intranasal	Drug permeability across BBB was enhanced.	[282]
TAT (AYGRKKRRQRRR) modified doxorubicin‐loaded liposomes	Doxorubicin (anti‐cancer agent)	Brain glioma treatment	Intravenous	The TAT‐modified liposome enhanced the therapeutic efficacy on brain glioma both in vitro and in vivo.	[283]
TAT‐poly(ethylene glycol) (PEG)‐b‐cholesterol (TAT–PEG‐b‐Chol)	Ciprofloxacin	Encephalitis	Intravenous	Improved in vitro cellular (ACBRI 376) uptake. NPs crossed the BBB and located around the cell nucleus of neurons	[284]
HAIYPRH peptide (T7)‐conjugated PEGylated liposomes (T7‐P‐LPs) loaded with ZL006 (T7‐P‐LPs/ZL006)	ZL006 (neuroprotectant)	Ischemic stroke therapy	Intravenous	This system can be used as an efficient targeted payload vehicle as a potential treatment of ischemic stroke.	[285]
Diketopiperazines (DKPs) (highly stable cyclic dipeptides) bearing peptides	Deliver a hexapeptide VQIVYK sequence	AD treatment	Intravenous	These DKP scaffolds vectors were capable of transporting peptides into the brain and inhibiting Tau aggregation in vivo in mice.	[286]
Angiopep‐2 functionalized with PEG‐PLA copolymers	Lipophilic drugs	Brain delivery	Intravenous	Angiopep‐modified PEG‐PLA micelle is a promising candidate to deliver lipophilic drugs.	[287]
Gold nanoparticles (GNP) functionalized with PEG and then conjugated to the shuttle Angiopep‐2 (GNR‐PEG‐Angiopep‐2)	Gold nanorod	CNS delivery	Intravenous	Enhancing the delivery of gold nanorod to the CNS by using Angiopep‐2	[288]
miniAp‐4 (H‐DapKAPETALD‐NH2) cyclic peptides (bee venom‐derived Apamin) shuttle peptide	Gold nanoparticle	Mice brain parenchyma	Intravenous	Resistant to proteases and capable to efficiently deliver diverse cargoes across the BBB Remarkable brain‐targeting capacity Noticeably less toxic and immunogenic than the native peptide	[289]
Polyester‐based NPs functionalized with 15F, and REG peptides	Paclitaxel (PTX)	Glioma therapy	Intravenous	According to the results, dual‐targeting drug delivery vehicle might have an excellent potential to be applied in clinical applications for glioma treatments.	[290]

## 5 Conclusion and Future Trends

In addition to damage to the brain tissue, TBI triggers neuroinflammation and oxidative stress, which accelerates neuronal cell death, hinders functional recovery, and damages the surrounding penumbra irreversibly. Due to the unfavorable microenvironment of TBI and limited drug penetration across the BBB, treating CNS injuries can be challenging. A new strategy is therefore urgently needed to provide physical support to the cells within and adjacent to injury sites while improving sustained drug release without causing immune responses or inflammation. In the past decade, SAP hydrogels have offered several advantages over other biomaterials and demonstrated great potential as implantable functional biomaterials for CNS injuries.

A comprehensive analysis of SAP hydrogels' physiochemical properties indicates that by adjusting properties such as 2D or 3D structure, stiffness, porosity, nanotopography, wettability, surface charge, and molecular presentation, a conducive environment can be created in the injured area by sustained delivery of inflammatory agents and regulation of stem cell host immune responses. This promise of utilizing nature's own programming code to develop side chain interactions that dictate nano‐architectures, creating molecularly functional building blocks, is appealing to bioengineers, clinicians and commercial bodies.

The recent cost reduction and scale up in manufacturing, compared to the costly production observed in the early 2000′s, has prompt the surge of clinical and pre‐clinical trials. Following the success of commercial chiral SAPs in the less clinically challenging cosmetic and wound‐healing fields, it is expected that SAP hydrogels will revolutionize the treatment of CNS injuries and diseases.

A limitation to overcome before we can see widespread adoption of SAP hydrogels in the clinic will be to ensure neuroinflammatory modulation. This effect must be spatiotemporally controlled to permit the initial paracrine signaling responses to recruit reactive astrocytes but limit the detrimental effect that reactive astrocyte overaccumulation might have. At the same time, the SAP must be designed (or have the capacity) to degrade at a rate that effectively matches tissue regeneration allowing for cells to mature, establish connections and form mature, functionally indistinct tissue while limiting glial scar formation. Despite the rich potential of this approach, given the high complexity involved, the SAP enabled structures remain under investigation, as each feature of the biomaterial intervention is interlinked, and modifications to one aspect may significantly impinge on another, leading to unforeseen outcomes. We must therefore continue to study their physiochemical properties and neuroinflammatory response in depth. This class of materials has only just begun to show its full potential for therapeutic use, and the coming decades will reveal its full range of applications.

## Conflict of Interest

The authors declare no conflict of interest.
